# Investigating the Viral Suppressor HC-Pro Inhibiting Small RNA Methylation through Functional Comparison of HEN1 in Angiosperm and Bryophyte

**DOI:** 10.3390/v13091837

**Published:** 2021-09-15

**Authors:** Neda Sanobar, Pin-Chun Lin, Zhao-Jun Pan, Ru-Ying Fang, Veny Tjita, Fang-Fang Chen, Hao-Ching Wang, Huang-Lung Tsai, Shu-Hsing Wu, Tang-Long Shen, Yan-Huey Chen, Shih-Shun Lin

**Affiliations:** 1 Institute of Biotechnology, National Taiwan University, Taipei 106, Taiwan; sanobar.neda@gmail.com (N.S.); pinchun.lin26@gmail.com (P.-C.L.); zoeypan00@gmail.com (Z.-J.P.); ryfang22@gmail.com (R.-Y.F.); venytjita97@gmail.com (V.T.); fun_0108@hotmail.com (F.-F.C.); yhcapple@gmail.com (Y.-H.C.); 2 The PhD Program for Translational Medicine, College of Medical Science and Technology, Taipei Medical University and Academia Sinica, Taipei 115, Taiwan; wanghc@tmu.edu.tw; 3 Graduate Institute of Translational Medicine, College of Medical Science and Technology, Taipei Medical University, Taipei 110, Taiwan; 4 Institute of Plant and Microbial Biology, Academia Sinica, Taipei 115, Taiwan; huanglungtsai@ntu.edu.tw (H.-L.T.); shuwu@gate.sinica.edu.tw (S.-H.W.); 5 Institute of Molecular and Cellular Biology, National Taiwan University, Taipei 106, Taiwan; 6 Center of Biotechnology, National Taiwan University, Taipei 106, Taiwan; shentl@ntu.edu.tw; 7 Department of Plant Pathology and Microbiology, National Taiwan University, Taipei 106, Taiwan; 8 Agriculture Biotechnology Research Center, Academia Sinica, Taipei 115, Taiwan

**Keywords:** sRNA, HEN1, methylation, viral suppressors, HEN1-HC-Pro interaction

## Abstract

In plants, HEN1-facilitated methylation at 3′ end ribose is a critical step of small-RNA (sRNA) biogenesis. A mutant of well-studied Arabidopsis HEN1 (AtHEN1), *hen1-1*, showed a defective developmental phenotype, indicating the importance of sRNA methylation. Moreover, *Marchantia polymorpha* has been identified to have a *HEN1* ortholog gene (Mp*HEN1*); however, its function remained unfathomed. Our in vivo and in vitro data have shown MpHEN1 activity being comparable with AtHEN1, and their substrate specificity towards duplex microRNA (miRNA) remained consistent. Furthermore, the phylogenetic tree and multiple alignment highlighted the conserved molecular evolution of the HEN1 family in plants. The P1/HC-Pro of the turnip mosaic virus (TuMV) is a known RNA silencing suppressor and inhibits HEN1 methylation of sRNAs. Here, we report that the HC-Pro physically binds with AtHEN1 through FRNK motif, inhibiting HEN1’s methylation activity. Moreover, the in vitro EMSA data indicates GST-HC-Pro of TuMV lacks sRNA duplex-binding ability. Surprisingly, the HC-Pro also inhibits MpHEN1 activity in a dosage-dependent manner, suggesting the possibility of interaction between HC-Pro and MpHEN1 as well. Further investigations on understanding interaction mechanisms of HEN1 and various HC-Pros can advance the knowledge of viral suppressors.

## 1. Introduction

The 21- to 24- nucleotide (nt) short interfering RNA (siRNA) and microRNA (miRNA) mediate RNA silencing via a sequence-specific targeting of messenger RNA (mRNA) either by RNA repression or cleavage [1]. In plants, the miRNA/miRNA* duplexes are processed from long hairpin precursor miRNA by Dicer-like 1 (DCL1), which produces miRNA with 2-nt overhangs at 3′-end. In contrast, the siRNA duplexes are generated from long-double-stranded RNA by DCL2, DCL3, and DCL4 [2]. Next, the miRNA and siRNA duplexes obtain 3′-end 2′-*O*-methylation by Hua-Enhancer 1 (HEN1) methyltransferase [3,4,5]. The methylated miRNA and siRNA duplexes are loaded into ARGONAUTE (AGO1) of RNA INDUCED SILENCING COMPLEX (RISC) for further RNA silencing regulation [1].

Unlike animals, both miRNA and siRNA of plants need to be methylated by HEN1. For this modification, siRNA was found to be competing with miRNA, and it is crucial as this 2′-OH of the 3′-end ribose upon methylation protects the small RNA (sRNA) from tailing and trimming effects [4,6,7]. The *hen1-1* null mutant shows heterogeneity at their 3′-end ribose, usually 1–6 uridine residues being added by terminal nucleotidyl transferases like HEN1 Suppressor1 (HESO1) and UTP: RNA URIDYLYLTRANSFERASE (URT1), where both the enzymes tend to have hierarchical as well as cooperative effects [4,5,7,8,9]. Moreover, *hen1-1* mutant caused the miRNA levels to reduce resulting in downregulation or inefficiency of the gene silencing pathway [4,7,10].

With the structural knowledge, the HEN1 of Arabidopsis comprises five different domains among which the double-stranded RNA-binding domains (dsRBD1 and dsRBD2) recognize the substrate RNA, the LCD domain, which contains a La motif binds the 3′ end of the RNA, the PPIase-like domain (PLD), which has structural similarity to FK506-binding proteins, and the methyltransferase domain (MTase), which measures the length of the sRNA and facilitates the 2′-*O*-methylation [11,12]. Besides plants, other species belonging to fungi and animals also have HEN1 orthologs performing similar functions of RNA methylation. In animals, the HEN1 orthologs do not methylate its miRNA; instead, the piwi-interacting RNA (piRNAs) and siRNA of animals, which are single-stranded RNA of 25- to 31-nt, obtain 3′-end 2′-*O*-methylation by the HEN1 [13,14,15]. Even if there is no RNA interference in the bacteria, the HEN1 ortholog has an even more interesting function here. The HEN1 participates in the RNA repair mechanism through the 3′-end 2′-*O*-methylation to protect the RNA from future damage [16].

*Marchantia polymorpha*, commonly known as liverwort, is a member of basal land plant lineage [17]. Due to its short life cycle, and other benefits, including ease of propagation and crossing, high transformation efficiency, small genome size, and lower genetic redundancy, *M. polymorpha* has become one of the most studied species of liverworts [18,19]. *M. polymorpha* contains a copy of the RNA silencing system, including DCL1, AGO1, and HEN1, etc. [20,21,22]. Our previous study demonstrated that the miRNAs were methylated in *M. polymorpha*; however, so far, there is no biological evidence to demonstrate the MpHEN1 activity [21].

Previous studies showed that the HC-Pro of potyvirus plays an important role in the suppression of RNA silencing [23,24,25,26,27]. The Arg of FRNK motif of HC-Pro (HC-Pro^R^) is critical for miRNA-mediated gene regulation and viral pathogenicity [23]. The Arg mutated to Lys (FKNK) on HC-Pro (HC-Pro^K^) abolished the suppression ability of the miRNA pathway and generated a mild strain for cross-protection [23]. We inserted a green fluorescent protein (GFP) gene in the genome sequence of the turnip mosaic virus (TuMV) infectious clone to generate TuGR recombinant wild type virus [28] and also produced the HC-Pro^K^ mutated mild strain of TuMV (TuGK) that delivered mild symptoms on Arabidopsis and *Nicotiana benthamiana* [23]. The transgenic Arabidopsis expressing the *P1/HC-Pro^R^* gene of TuGR (*P1/HC-Pro^R^* plant) showed a serrated and curled leaf phenotype [23]. In contrast, the transgenic Arabidopsis expressing *P1/HC-Pro^K^* gene (*P1/HC-Pro^K^* plant) showed a normal developmental phenotype [23]. The further investigated results demonstrated that the HC-Pro^K^ lost the ability to suppress the miRNA pathway [23,29].

By using molecular, biochemical, and structural biology approaches, we studied the Arabidopsis HEN1 (AtHEN1) as well as *M. polymorpha* HEN1 (MpHEN1) characteristics in this study. We demonstrated the in vitro and in vivo methyltransferase activity of AtHEN1 and MpHEN1. The HC-Pro^R^ of TuMV inhibits the AtHEN1 methylation activity both in vivo as well as in vitro through the FRNK motif by physical interaction, but the HC-Pro^R^ does not have sRNA-binding activity. Surprisingly, the HC-Pro^R^ was also found to suppress the MpHEN1 activity. Additionally, we generated AtHEN1 and MpHEN1 antibodies for the protein detection. The in vitro methylation results showed that both HEN1s have duplex RNA substrate specificity. Through the amino acids sequence alignment and the architectural analysis, several critical metal-binding residues and S-adenosyl methionine (SAM) residues remained conserved between AtHEN1 and MpHEN1. Together, these data demonstrate the significance of sRNA methylation in both angiosperm and bryophyte. Additionally, the HEN1 studies can be exploited to understand the plant silencing system and HC-Pro suppression mechanism.

## 2. Materials and Methods

### 2.1. Plant Growth Conditions

The seeds of *Arabidopsis thaliana* ecotype Columbia (Col-0), *P1/HC-Pro^R^*, and *P1/HC-Pro^K^* plants [23] and *hen1-8/heso1-1* double mutant [8] were used in this study. For the overexpressing *HEN1* in Col-0 plant, the 35S promoter-driven *HA-AtHEN1* gene (35Spro:*HA-AtHEN1*) constructed was introduced into Col-0 plants to generate *HA-AtHEN1* plants. Furthermore, the 35S promoter-driven *P1/HC-Pro^R^* gene and 35Spro:*HA-AtHEN1* were introduced into Col-0 to generate the *P1/HC-Pro^R^*/*HA-AtHEN1* plant, and the 35S promoter-driven *P1/HC-Pro^K^* gene and 35Spro:*HA-AtHEN1* were introduced into Col-0 to generate the *P1/HC-Pro^K^*/*HA-AtHEN1* plant. The seeds were plated on Murashige and Skoog (MS) medium upon surface sterilization. Appropriate antibiotic MS plates were used according to the transgenic resistant lines. The plants were kept in a growth room with 16 h light/8 h darkness, 20 to 25 °C.

*Marchantia polymorpha* Takaragaike-1 (Tak-1, male accession) and Takaragaike-2 (Tak-2, female accession) were used as wild types. Either *M. polymorpha* gemma or thallus were grown and maintained on half-strength Gamborg’s B5 medium containing 1% agar and MES 2-(N morpholino) ethanesulfonic.

### 2.2. Plasmid Construction

We synthesized the codon-optimized full-length of the *AtHEN1* gene (2826 bp) and *MpHEN1* gene (3053 bp) (GeneDireX, Inc., Taoyuan, Taiwan), and cloned it into the pET28 vector to generate pET28-syn-AtHEN1 and pET28-syn-MpHEN1, respectively. For cloning the MTase domain of *AtHEN1*, the pET28-syn-AtHEN1 plasmid was used as a template for MTase domain amplification by KOD with P-syn-MTase (5′-CAGCCATATGAGCGAGGAACGTATGGAAGCGGCGTTCTTT-3′)/M-syn-MTase (5′-GGTGCTCGAGCAGGTCGGTCTTCTTCTTCTCAACATCTTC-3′) primer set. The amplified MTase domain DNA fragment was cloned into pET28a to generate pET28-syn-AtMTase. For the *HC-Pro* cloning, we synthesized the codon-optimized *HC-Pro^R^* and *HC-Pro^K^* genes and cloned them into pGEX vector to generate pGEX-HC-Pro^R^ and pGEX-HC-Pro^K^ plasmids.

### 2.3. The Recombinant Protein Purification

The pET28-syn-AtHEN1, pET28a-syn-AtMTase, and pET28-MpHEN1 plasmids were respectively transferred into *E. coli.* BL21 strain for recombinant protein expression. The bacteria pellets were collected from 400 mL bacterial culture with 0.125 mM Isopropyl, and β-d-1-thiogalactopyranoside (IPTG) induction was lysed with 150 mL of lysis buffer (50 mM Tris-HCl, pH 8.0, 300 mM NaCl, 1 mM DTT, and 10 mM imidazole) with freshly prepared 1 mM phenylmethylsulfonyl fluoride (PMSF). The recombinant proteins were purified with 1 mL HisTrap column (GE Healthcare, Chicago, IL, USA) by FPLC (GE Healthcare, Chicago, IL, USA). The collected fractions were checked on sodium dodecyl sulfate-polyacrylamide gel electrophoresis (SDS-PAGE) and dialyzed twice with dialysis buffer (10 mM Tris-HCl, pH 8.0, 100 mM NaCl, 1 mM DTT, 0.1 mM EDTA, 2 mM MgCl_2_). For the purification of GST-HC-Pro^R^ and GST-HC-Pro^K^, the pGEX-HC_pro^R^ and pGEX-HC_pro^K^ plasmids were transformed into *E. coli.* BL21 strain and purified using the GSTrap FF column (GE Healthcare, Chicago, IL, USA).

### 2.4. The α-AtHEN1 and α-MpHEN1 IgG Production

For AtHEN1 antibody production, the his-AtMTase recombinant protein was used as an antigen. For α-MpHEN1 antibody production, the full length of his-MpHEN1 was used as antigen. New Zealand rabbit was immunized with 1 mg of recombinant protein once a week for a month. The recombinant protein with Freund’s complete adjuvant (1:1 *v*/*v*) was injected in the first week and then Freund’s incomplete adjuvant was used for the rest of 3 weeks. Sera collected from the 5th week until the 8th week and the last week’s blood were subjected to IgG purification where 10 mL of antiserum mixed with 10 mL of 1× PBS buffer (137 mM NaCl, 2.7 mM KCl, 8.1 mM Na_2_HPO_4_, 1.5 mM KH_2_PO_4_, pH 7.4, 1:1 *v*/*v*) were loaded into a HiTrap Protein A column (GE Healthcare, Chicago, IL, USA). After the binding, the column was connected to the FPLC machine and was washed using 20 mM phosphate buffer (7.8 mM NaH_2_PO_4_, 12.2 mM Na_2_HPO_4_), and then the IgG was eluted using 0.1 M citric acid, pH 3.0 buffer. The protein fractions were collected with 200 µL 1M Tris-HCl, pH 8.0, and dialyzed using a 1× PBS buffer.

### 2.5. Western Blotting

The soluble protein was extracted from fourteen-day-old seedlings with a 2× sampling buffer (2% SDS, 10% glycerol, 1% β-mercaptoethanol, 0.005% bromophenol blue, 50 mM Tris-HCl, pH 6.8), cooked at 100 °C for 10 min and then kept on ice for at least 5 min. The samples were applied to SDS-PAGE and next blotted onto methanol-pretreated polyvinylidene difluoride (PVDF) membrane (GE Healthcare, Chicago, IL, USA). The PVDF membrane was incubated with primary antibody overnight shaking at 4 °C. The membrane was washed and then subjected to commercial HPR-conjugated anti-rabbit IgG (GE Healthcare, Chicago, IL, USA) as the secondary antibody. After 2 h of incubation, the membrane was washed again, and immunostained proteins were stained using enhanced chemiluminescence (ECL) Western blot detection reagent (PerkinElmer, Waltham, MA, USA) and exposed with X-ray film.

### 2.6. In Vitro and In Vivo HEN1 Methylation Assay

The single-stranded synthetic miR159a was annealed using 5× annealing buffer (300 mM KCl, 30 mM HEPES, pH 7.5, 1 mM MgCl_2_) for 5 min at 95 °C and then allowed to cool to room temperature. A total of 0.4 ng of ds-syn-miR159 was then subjected to a methylation process with 8 µL of purified his-AtHEN1 or his-MpHEN1 protein, 10 µL NEB Cutsmart buffer, and 3.2 mM SAM. For the in vivo methylation assay, the wild-type Col-0 was infected with TuGR (severe strain) and TuGK (milder strain). After 10 days post-inoculation (dpi), the infected tissues were used for total RNA extraction. The RNA was quantified using a spectrophotometer, and 30 µg/10 µL was used for the experiment.

### 2.7. β-Elimination

The methylation activity of HEN1 was studied using the periodate oxidation method [4]. The reaction product was treated with sodium periodate for oxidation in darkness at room temperature for an hour and was stopped using 1/10 volume of glycerol for about 30 min. RNA was then precipitated at 4 °C for 10–15 min at 13,000 rpm. β-elimination was carried out by dissolving the precipitated RNA in 100 µL of 0.055 M borax/boric acid/NaOH (pH 9.5) and incubated at 45 °C for 90 min. RNA was precipitated again the same way as before and then subjected to northern blot analysis where the RNA was separated over 20% polyacrylamide gel containing 8 M urea and hybridized with ^32^P labeled As-miR159a (5′-TAGAGCTCCCTTCAATCCAAA-3′). The signal of miR159a on the membrane was exposed with the X-ray film.

### 2.8. In Vitro Pull-Down and In Vivo IP

For in vitro pull-down, 2 μg of bait (GST-HC-Pro^R^ or GST-HC-Pro^K^) and 2 μg prey (his-AtHEN1) proteins were added to 1 mL of binding buffer (50 mM Tris-HCl, pH 7.5, 100 mM NaCl, 0.2% glycerol, 0.6% Triton X-100, 0.5 mM β-mercaptoethanol). After incubation at 25 °C for 2 h, the reaction mixture was further incubated with Glutathione Sepharose 4B resin (GE Healthcare, Chicago, IL, USA) beads for 2 h before being washed six times with the washing buffer (50 mM Tris-HCl, pH 7.5, 100 mM NaCl, 0.6% Triton X-100). Pulled-down proteins were analyzed by western blotting using α-His or α-GST antibodies.

For in vivo IP, 1-week-old (1 g) *P1/HC-Pro^R^*/*HA-AtHEN1* or *P1/HC-Pro^K^*/*HA-AtHEN1* seedlings were homogenized with 1 mL IP buffer (25 mM Tris-HCl, pH 7.5, 150 mM NaCl, 1 mM EDTA, 5% glycerol, 1% NP-40), followed by centrifuging for 10 min at 4 °C. Notably, the IgG of α-HA was used for the in vivo IP of this study. IP was performed by a mix of cleaned Protein A-Agarose beads (50 μL suspension per IP reaction) (Santa Cruz, Dallas, TX, USA), IgG (30 μL per IP reaction), and the lysate. The IP reaction was set at 4 °C with gentle mixing for 3 h. The tube was centrifuged at 300× *g* to pull-down the beads and the unspecific binding was washed with 0.3 mL IP buffer twice and suspended with 0.3 mL IP buffer. The IP elutes were used for Western blotting or small RNA extraction.

### 2.9. EMSA

The electrophoresis mobility shift assay (EMSA) protocol was performed as described by Rio [30]. The ^32^P-labeled synthetic miRNA/miRNA* duplex was incubated with purified protein (1 μg) in a binding buffer (40 mM Tris-HCl, pH 8.0, 30 mM KCl, 1 mM MgCl_2_, 0.01% NP40, 1 mM DTT) for 1 h at 4 °C. The 4.2% native polyacrylamide gel (acrylamide:bisacryladmide = 80:1) was used for the EMSA assay. The gel was exposed to X-ray film at −80 °C for further analysis.

### 2.10. MpHEN1 Structure Modeling

The protein model for MpHEN1 was proposed by using SWISS-MODEL (www.expasy.org/resources/swiss-model, accessed on 26 June 2021). The accuracy of the resulting model was evaluated by GMQE (Global Model Quality Estimation) and QMEAN (Qualitative Model Energy Analysis) parameters [31]. The GMQE and QMEAN scores (0.60 and 4.09) of the MpHEN1 model are close to the indicator values recommended on the SWISS-MODEL website. This means that the presented model can be referenced. We applied the default setting for all parameters within the algorithms without any modifications.

### 2.11. Sequence Identity, Similarity, and Phylogenetic Analysis

The pairwise sequence identity and similarity of the MTase domain of HEN1 and HEN1 orthologs were evaluated using SIAS (imed.med.ucm.es/Tools/sias.html, accessed on 31 July 2021). Additionally, the different domains of AtHEN1 were analyzed for their amino acid identity and similarity using an Emboss needle (www.ebi.ac.uk/Tools/psa/emboss_needle/, accessed on 24 June 2021).

HEN1 and HEN1-like orthologs were selected for phylogenetic tree construction according to [32,33]. Protein sequences were identified through BLAST and searched from Phytozome v12.1 (phytozome.jgi.-doe.gov, accessed on 31 July 2021), Gymno PLAZAv1.0 (bioinformatics.psb.ugent.be/plaza/versions/gymno-plaza, accessed on 31 July 2021), the algae genome project (www.plantmorphogenesis.bio.titech.ac.jp/~algae_genome_project/klebsormidium, accessed on 31 July 2021), TAIR (www.arabidopsis.org, accessed on 31 July 2021), and NCBI (www.ncbi.nlm.nih.gov, accessed on 31 July 2021). Multiple sequences were aligned with CLUSTAL W v2.1 (www.genome.jp/tools-bin/clustalw, accessed on 31 July 2021). Phylogenetic reconstructions were performed using the function “build” of ETE3 v3.1.1 [34] as implemented on the GenomeNet (www.genome.jp/tools/ete, accessed on 31 July 2021). The tree was constructed using fasttree with slow NNI and MLACC = 3 (to make the maximum-likelihood NNIs more exhaustive) [35]. Values at nodes are SH-like local support. We applied the default setting for all parameters within the algorithms without any modifications.

## 3. Results

### 3.1. HEN1 Antibody Production and Sensitivity Evaluation

**Figure 1:**
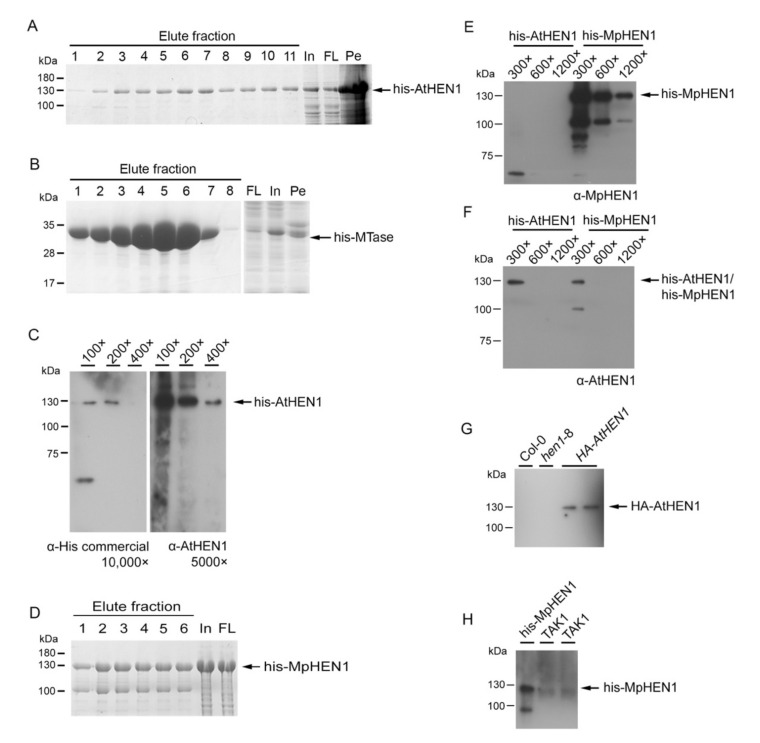
α-AtHEN1 and α-MpHEN1 antibodies production and sensitivity analysis. (**A**) The SDS-PAGE analysis for the eluted fractions of the recombinant full-length his-AtHEN1. The band corresponding to 130 kDa showed the presence of the expected protein. (**B**) SDS-PAGE analysis for the eluted his-MTase. The prominent band at 34 kDa depicted its expression. (**C**) The sensitivity evaluation of α-AtHEN1 (5000× dilution) that compared with his-monoclonal antibody (10,000× dilution). (**D**) The SDS-PAGE analysis for the eluted fraction of the recombinant full-length his-MpHEN1. In, input. FL, flow-through, Pe, pellet. The evaluation of antibody cross-reaction between α-AtHEN1 (**E**) and α-MpHEN1 (**F**) antibodies at different dilutions of his-AtHEN1 and his-MpHEN1. Both antibodies were diluted at 10,000×. (**G**) The endogenous AtHEN1 detection for α-AtHEN1 antibody. WT, Col-0. *hen1-8*, the *HEN1* mutant. *HA-AtHEN1*, the *HA-AtHEN1* plant. (**H**) The endogenous MpHEN1 detection for α-MpHEN1 antibody. His-MpHEN1, the recombinant MpHEN1. TAK1, the WT of male *M. polyporpha*.

To investigate the HEN1 characterization, we generated an AtHEN1 antibody for the detection of endogenous HEN1. First of all, we purified the full-length of recombinant his-AtHEN1 through *E. coli* BL21. The result indicated that approximately 130 kDa his-AtHEN1 can be detected on the coomassie blue staining page, and the size of protein was consistent with the predicted size (Figure 1A). However, we realized that the amount of purified his-AtHEN1 for rabbit immunization might not be sufficient (Figure 1A). Hence, we evaluated the expression of all five different domains of AtHEN1 for evaluating the best domain for expression and immunization. We tested the recombinant domain expressions for dsRBD1 (1–86 aa), LCD domain (95–357 aa), dsRBD2 (387–500 aa), PLD (535–683 aa), and MTase domain (690–940 aa). The result showed that the his-MTase can generate abundant recombinant protein (Figure 1B). Therefore, the recombinant his-MTase was used for immunization to generate the antibody. The different dilutions of his-AtHEN1 were used to evaluate the antibody efficiency, and the result showed that the antibody can detect the his-AtHEN1 at 130 kDa position same as the corresponding signal detected with using commercial α-His antibody (Figure 1C), which is consistent with the predicted molecular weight of the protein. Therefore, this antibody generated by his-MTase can be claimed as an α-AtHEN1 antibody.

Next, we generated the MpHEN1 antibody with full-length his-MpHEN1 to test whether full-length recombinant HEN1 can be a good antigen for antibody generation (Figure 1D). The full-length generated α-MpHEN1 antibody detected the his-MpHEN1 even at 1200× dilution and no cross-detection for his-AtHEN1 (Figure 1F). These data suggested that the full-length his-MpHEN1 antigen has more sequence specificity in antibody generation. Interestingly, the α-AtHEN1 can detect the his-AtHEN1 and his-MpHEN1 at 300× dilution (Figure 1F). Furthermore, we found 42% amino acid identity between AtHEN1 and MpHEN1 (Figure 7A), which could be contributing to the ability of α-AtHEN1 antibody to show cross-reactivity toward his-MpHEN1.

Next, we evaluated the ability of α-AtHEN1 in endogenous HEN1 detection. The results indicated that α-AtHEN1 can detect exogenous AtHEN1 in the transgenic Arabidopsis expressing the *HA-AtHEN1* gene (*HA-AtHEN1* plant), whereas no signal was detected in Col-0 and *hen1-8* mutant, indicating that α-AtHEN1 might have less efficiency in endogenous AtHEN1 detection (Figure 1G). Similarly, α-MpHEN1 also cannot detect the endogenous MpHEN1 protein in TAK1 (Figure 1H).

### 3.2. The Recombinant HEN1 Methylation Ability and Substrate Specificity

**Figure 2:**
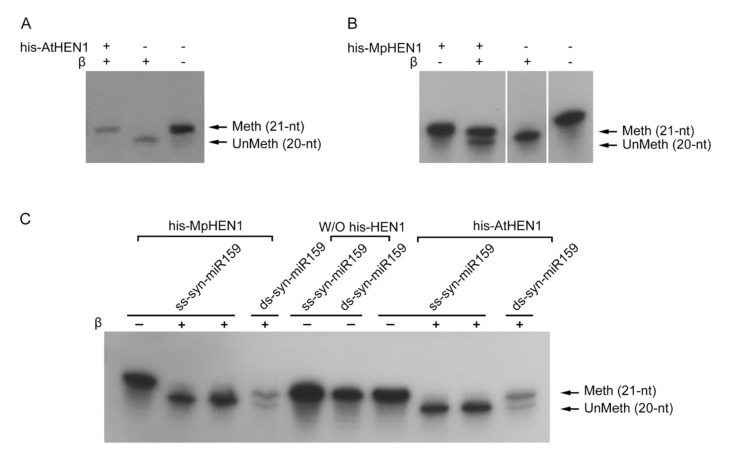
In vitro methylation activity and substrate specificity for his-AtHEN1 and his-MpHEN1. The his-AtHEN1 (**A**) and his-MpHEN1 (**B**). In vitro methyltransferase activity assay. Meth, the 21-nt position represents the methylated miRNA. UnMeth, the 20-nt position represents the unmethylated miRNA. (**C**) The substrate specificity evaluation of AtHEN1 and MpHEN1.

To further evaluate the HEN1 characteristics, the methylation activity and substrate specificity for his-AtHEN1 were performed using an in vitro assay. In the presence of AtHEN1, all syn-miR159a samples were methylated, represented by the signal at 21-nt position (upper band), while syn-miR159a remained unmethylated, and its signal was represented by 20-nt position (lower band) in the absence of his-AtHEN1 (Figure 2A). Similarly, the in vitro methylation of his-MpHEN1 showed 67% methylated syn-miR159a, indicating the role of MpHEN1 in miRNA methylation (Figure 2B).

Previous studies demonstrated that HEN1 orthologs in different species have diverse RNA substrates. Some orthologs restrict only for single-stranded substrate while others choose to methylate duplex RNA [5,15,36]. Therefore, we evaluated the substrate for AtHEN1 and MpHEN1. We used single-stranded syn-miR159 (ss-syn-miR159) and double-stranded syn-miR159 (ds-syn-miR159) as substrates for the in vitro HEN1 methylation assay. The results showed that his-AtHEN1 and his-MpHEN1 can methylate ds-syn-miR159, but not ss-syn-miR159, suggesting that both HEN1 in bryophyte and angiosperm have substrate specificity with miRNA duplex methylation (Figure 2C).

### 3.3. HC-Pro Suppressor of TuMV Inhibits HEN1 Activity

**Figure 3:**
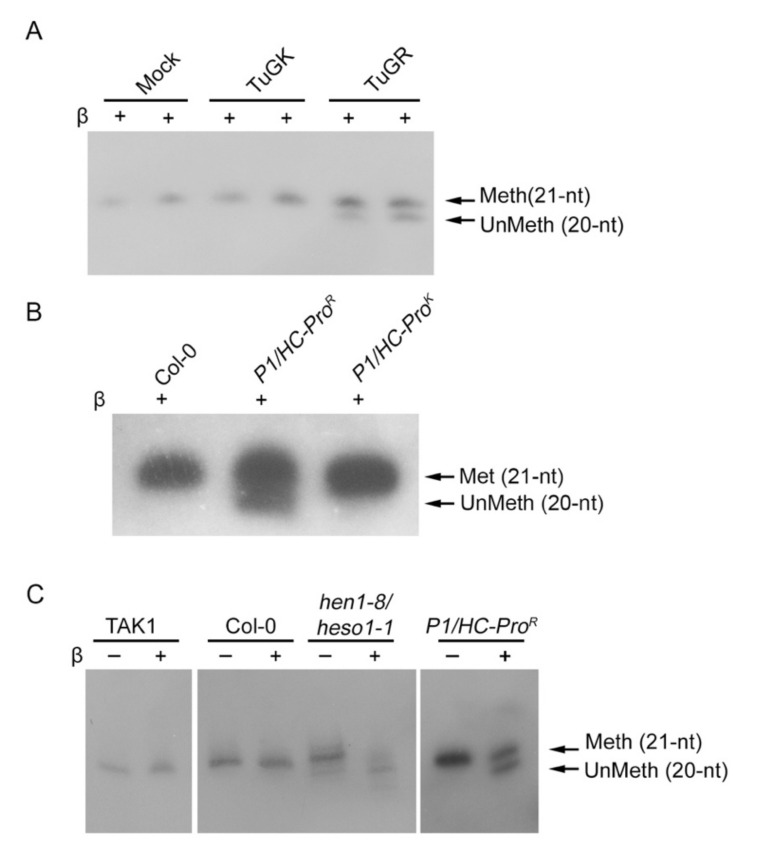
The HC-Pro-mediated HEN1 activity inhibition. (**A**) The in vivo miR159 methylation status in wild-type TuMV (TuGR) and TuMV mutant (TuGK)-infected Col-0. (**B**) Evaluation of miRNA methylation status in *P1/HC-Pro^R^* and *P1/HC-Pro^K^* plants. The miRNA methylation status of 1-week-old plants was examined by oxidation/β-elimination, followed by small-RNA Northern blotting. (**C**) The in vivo miR166a methylation status in various mutant and transgenic plants. TAK1, the wild-type *M. polymorpha*, Col-0, wild-type Arabidopsis. *hen1/heso1* mutant, the *HEN1*, and *HESO1* double mutant. *P1/HC-Pro^R^*, *P1/HC-Pro^R^* plant.

Yu et al. (2006) demonstrated that the miRNA methylation is inhibited in transgenic Arabidopsis expressing the *HC-Pro* gene of TuMV (*HC-Pro* plant), implying that HC-Pro might suppress the HEN1 activity [29]. Thus, we evaluated the status of endogenous miRNA methylation in 14 dpi of TuGR and TuGK-infected Col-0 plants. The β-elimination results depicted that the miR159 in the mock and TuGK-infected plants remained 100% methylated while in the case of TuGR, the methylation state was reduced to 65% (Figure 3A), suggesting the wild type of HC-Pro can inhibit the HEN1 methylation in miRNA. Similar results also showed in *P1/HC-Pro^R^* and *P1/HC-Pro^K^* plants. The *P1/HC-Pro^R^* plant inhibited miRNA methylation, whereas the *P1/HC-Pro^K^* plant had normal miRNA methylation like Col-0 plants (Figure 3B).

The β-elimination assay showed both TAK1 and Col-0 have fully methylated miRNA in vivo, indicating that the MpHEN1 might serve an identical methylation function in *M. polymorpha* as it does in Arabidopsis (Figure 3C). We found a smear of lower molecular weight bands in *hen1/heso1* mutant, indicating truncation or degradation of the miRNAs being facilitated by exoribonucleases such as small RNA-degrading nuclease (SDN) (Figure 3C) [37]. The *P1/HC-Pro^R^* plant showed 47% partial inhibition of miRNA methylation (Figure 3C). Collectively, the observations suggest that the basic behavior of HEN1s in *M. polymorpha* and Arabidopsis is comparable.

### 3.4. HC-Pro^R^ Binds HEN1 to Impair miRNA Methylation

**Figure 4:**
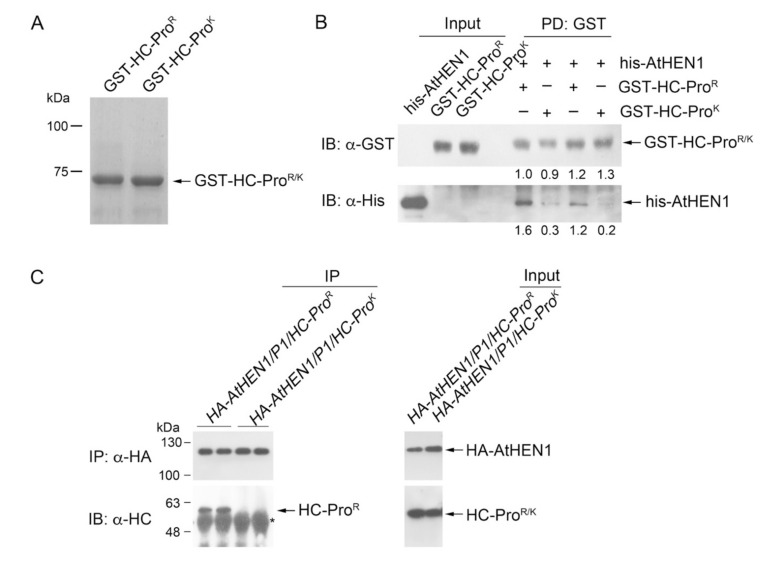
In vitro and in vivo HC-Pro^R^ and HEN1 interaction. (**A**) The SDS-PAGE for GST-HC-Pro^R/K^ and the band at around 75 kDa shows the expected protein size. (**B**) In vitro pull-down assay for the evaluation of the GST-HC-Pro^R^, GST-HC-Pro^K^, and his-HEN1 interaction. (**C**) In vivo co-IP assay to examine the binding activity between GST-HC-Pro^R^ or GST-HC-Pro^K^, and HA-HEN1. * Heavy chain of antibody.

The purified recombinant GST tagged HC-Pro^R/K^ showed good expression (Figure 4A). In vitro physical interaction data showed that GST-HC-Pro^R^ presented a 5- to 6-fold higher his-HEN1 pull-down signal than GST-HC-Pro^K^ (Figure 4B, lower panel). *P1HC-Pro^R^*/*HA-AtHEN1* and *P1HC-Pro^K^/HA-AtHEN1* plants were subsequently generated for in vivo co-immunoprecipitation (co-IP) assays. The co-IP data indicated that HC-Pro^R^ physically interacts with HA-AtHEN1, whereas no interaction signal of HC-Pro^K^ and HA-AtHEN1 could be detected (Figure 4C). These data demonstrated that the Arg of the FRNK motif is necessary for high AtHEN1-binding affinity.

**Figure 5:**
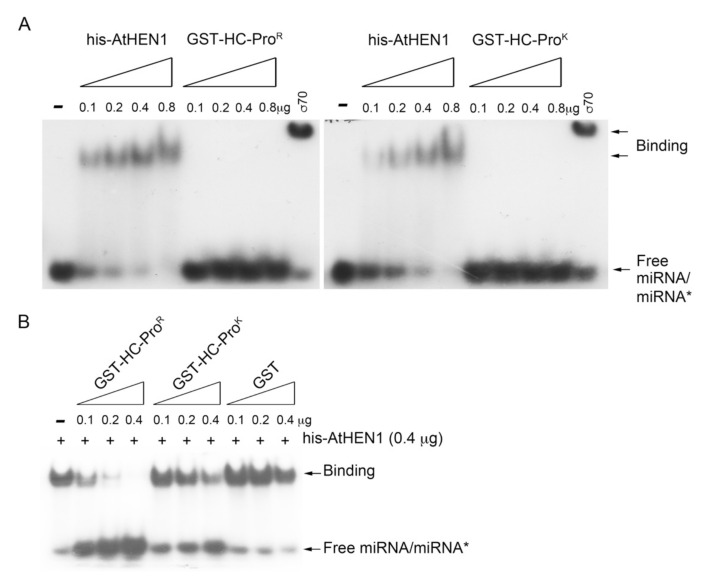
The miRNA/miRNA* and HC-Pro^R/K^-binding assay. (**A**) EMSA to examine the miRNA/miRNA*-binding ability of his-AtHEN1 and GST-HC-Pro^R^ or GST-HC-Pro^K^. (**B**) In vitro competition assay.

The EMSA demonstrated strong miRNA duplex-binding signals for the his-AtHEN1 protein. Neither HC-Pro^R^ nor HC-Pro^K^ showed a shift in the miRNA/miRNA* signals (Figure 5A). Sigma 70 (an RNA-binding protein) was used as a positive control for RNA binding (Figure 5A). To further elucidate the relationship between HC-Pro^R^ and HEN1 in terms of RNA binding, we examined whether HC-Pro^R^ influences HEN1-miRNA duplex binding. The EMSA results showed that HC-Pro^R^ strongly inhibited the miRNA duplex-binding activity of HEN1 in a dose-dependent manner (Figure 5B). In contrast, only high levels of HC-Pro^K^ (0.4 μg) interfered slightly with the miRNA/miRNA*-binding ability of HEN1 (Figure 5B), suggesting that HC-Pro^K^ cannot bind AtHEN1 efficiently and interfere with the RNA-binding activity of AtHEN1. Indeed, an in vitro pull-down assay demonstrated that HC-Pro^K^ still exhibited a slight interaction (0.2- to 0.3-fold) with AtHEN1 (Figure 4B). In summary, HC-Pro^R^ inhibits the methyltransferase activity of AtHEN1 toward miRNA/miRNA* through physical interaction to prevent HEN1-miRNA/miRNA* binding.

### 3.5. HC-Pro^R^ Inhibits HEN1 Methyltransferase Activity In Vitro

**Figure 6:**
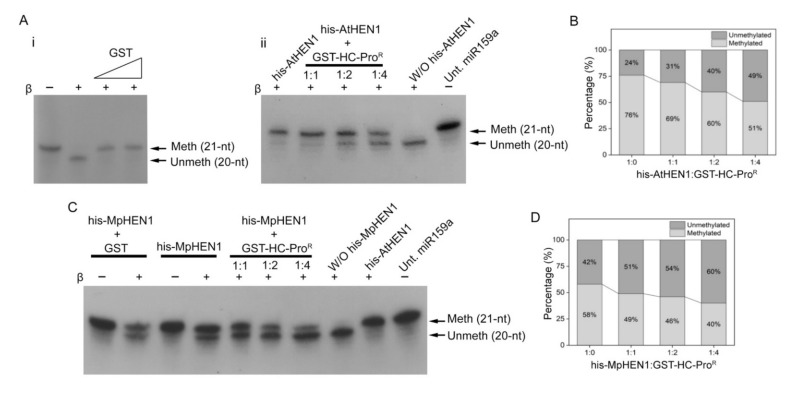
The in vitro HEN1 activity inhibition assay. (**A**) The in vitro HC-Pro^R^-mediated his-AtHEN1 inhibition assay. The GST was used as a negative control (i). The different ratios of his-AtHEN1 vs. GST-HC-Pro^R^ were performed in the HEN1 inhibition assay (ii). (**B**) Graph depicting quantification of GST-HC-Pro^R^-mediated his-AtHEN1 inhibition. (**C**) The in vitro HC-Pro^R^-mediated his-MpHEN1 inhibition assay. The different ratios for his-MpHEN1 vs. GST-HC-Pro^R^ were performed in the HEN1 inhibition assay. (**D**) Graph depicting quantification of GST-HC-Pro^R^-mediated his-MpHEN1 inhibition. Meth, the 21-nt position of methylated miRNA. UnMeth, the 20-nt position of unmethylated miRNA. Untreated miR159a is abbreviated as Unt. miR159a.

Next, we performed the in vitro HC-Pro-mediated HEN1 inhibition assay. The results showed that increasing the GST-HC-Pro^R^ to his-AtHEN1 ratio correlates positively with its inhibitory effect on the methylation property of his-AtHEN1 (Figure 6A,B). Surprisingly, GST-HC-Pro^R^ also inhibited the MpHEN1 activity in a dosage-dependent manner (Figure 6C,D). Arabidopsis is among the host plants for TuMV and hence its silencing suppressor HC-Pro can be expected to show an inhibitory effect against methylation [29,38]. On the other hand, *M. polymorpha*, a basal land plant, is not a host for TuMV, but surprisingly the GST-HC-Pro^R^ is able to repress the his-MpHEN1 activity (Figure 6C), suggesting that the structural similarities between both HEN1s could be the reason allowing HC-Pro^R^ to interact and inhibit. In summary, the in vitro results implied that HC-Pro^R^ alone without P1 were capable of facilitating the miRNA methylation suppression activity.

### 3.6. Functional Domain Comparison between AtHEN1 and MpHEN1

**Figure 7:**
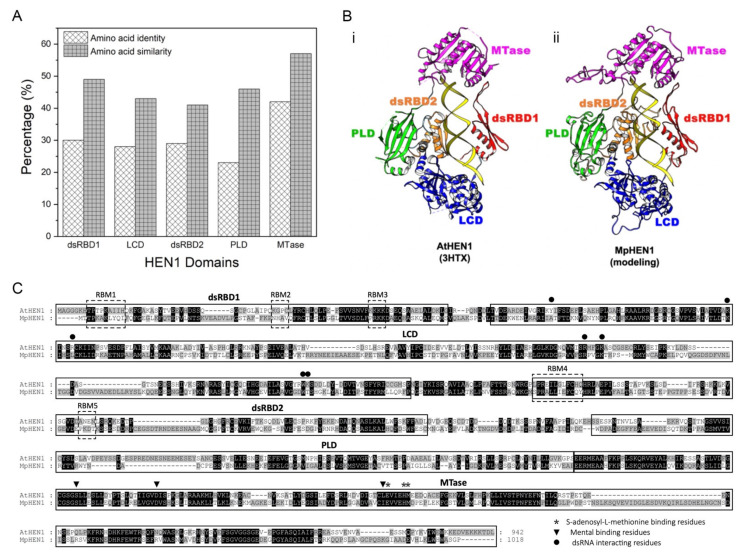
AtHEN1 and MpHEN1 structural analysis. (**A**) The domain comparison between AtHEN1 and MpHEN1. The Y-axis represents the percentage of similarity and identity. The X-axis represents the domains of HEN1. (**B**) Structural comparison between (i) AtHEN1 and (ii) MpHEN1 model. The dsRBD1, LCD, dsRBD2, PLD, and MTase domains on the AtHEN1 (PDB number: 3HTX) and MpHEN1 models are highlighted by red, blue, orange, green, and magenta, respectively. (**C**) Amino acid sequence alignment for AtHEN1 and MpHEN1. The five domains are highlighted by a box based on AtHEN1 structure studies and compared with the MpHEN1 sequence. The arrowheads, asterisks, and dots indicate the SAM-binding residues, metal-binding residues, and double-stranded RNA-interacting residues, respectively.

The AtHEN1 protects miRNAs and siRNAs against HESO1/URT1-1-mediated degradation [39]. Our study demonstrated that MpHEN1 tends to facilitate a similar function as well by methylating its miRNA at 3′ end ribose. We compared the amino acid identity and similarity for five domains (dsRBD1, LCD, dsRBD2, PLD, and MTase) between AtHEN1 and MpHEN1 (Figure 7A). The dsRBD1 domain between AtHEN1 and MpHEN1 showed 30% identity and 49% similarity, respectively (Figure 7A). LCD, RBD2, and PLD domains showed 23–29% identity and 41–46% similarity between AtHEN1 and MpHEN1 (Figure 7A). In addition, the MTase domain has the highest conservation between AtHEN1 and MpHEN1 that showed 42% identity and 57% similarity (Figure 7A).

Moreover, we compared the AtHEN1 structure (3HTX) [11] with the proposed MpHEN1 model, and the result indicates that MpHEN1 contains the same functional domains of AtHEN1 and may assume similar functional roles in *M. polymorpha* (Figure 7B). Furthermore, in a pairwise amino acid alignment of AtHEN1 and MpHEN1, there are five RNA-binding motifs (RBM1 to RBM5) present on two dsRBDs of AtHEN1 (Figure 7C). However, except RBM4, the other four out of five are not conserved in MpHEN1 (Figure 7C). In addition, we detected that five out of seven dsRNA-interacting residues on the LCD of AtHEN1 are conserved in MpHEN1 (Figure 7C). Moreover, three SAM-binding residues and three metal-binding residues are highly conserved in AtHEN1 and MpHEN1 (Figure 7C). However, critical functional residues in the LCD and MTase domains are highly conserved in AtHEN1 and MpHEN1. Finally, all of these results suggest that MpHEN1 might have similar functionality to AtHEN1.

### 3.7. Conservation of HEN1 Orthologs in Plant Species

**Figure 8:**
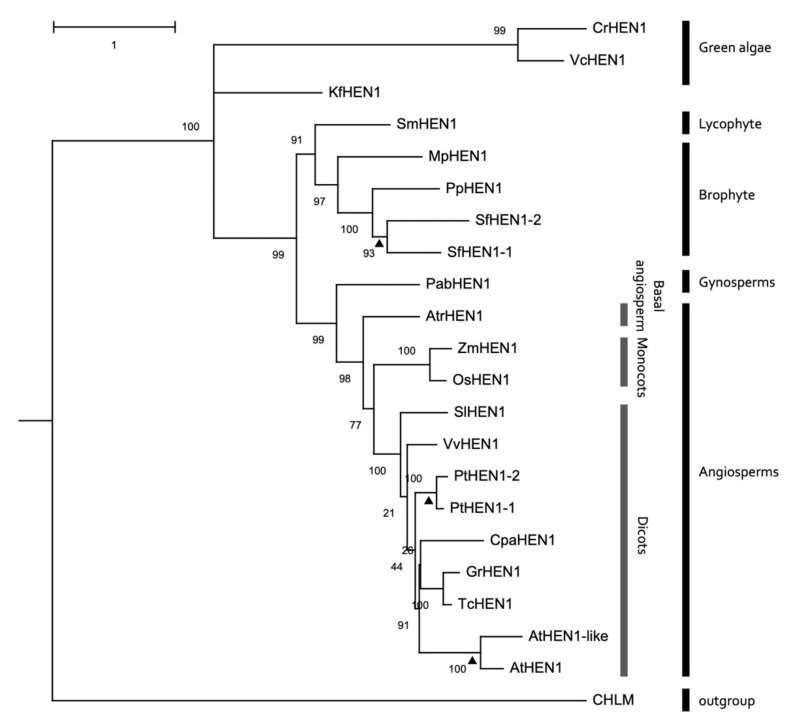
Phylogenetic tree of HEN1 and HEN1 orthologs of different species of plant kingdom. Maximum likelihood (ML) tree of 22 HEN1 orthologs of green plants. MAGNESIUM-PROTOPORPHYRIN IX METHYLTRANSFERASE (CHLM) of Arabidopsis was used as the outgroup. Abbreviation of species names: Sm, *Selaginella moellendorffii* (Smoe109211); At, *Arabidopsis thaliana* (AT4G20910); *Arabidopsis thaliana* (AT4G20920); Os, *Oryza sativa* (BAJ16352); Atr, *Amborella trichopoda* (evm_27.model.AmTr_v1.0_scaffold00092.83); Pab, *Picea abies* (PAB00043215); Mp, *Marchantia polymorpha* (Mp3G16010); Pp, *Physcomitrella patens* (Pp3c1_60V3.4); Kf, *Klebsormidium flaccidum* (kfl00033_0220_v1.1); Cr, *Chlamydomonas reinhardtii* (Cre03.g191200); Vc, *Volvox carteri* (Vocar.0035s0143.1); Zm, *Zea mays* (GRMZM2G107457_T01); Sf, *Sphagnum fallax* (Sphfalx0066s0042.1, Sphfalx0001s0228.1); Sl, *Solanum lycopersicum* (Solyc02g070030.2.1); Pt, *Populus trichocarpa* (Potri.001G465500, Potri.011G163600); Gr, *Gossypium raimondii* (Gorai.010G144100); Tc, *Theobroma cacao* (Thecc1EG026937); Cpa, *Carica papaya* (evm.model.supercontig_166.41), Vv, *Vitis vinifera* (GSVIVG01021670001) and *Arabidopsis thaliana*, CHLM (AT4G25080). The bootstrap values are shown above the branches at the nodes. Arrowheads indicate duplicated events.

A phylogenetic tree was constructed to elucidate the relationships among HEN1 and HEN1 orthologs from species of green plants. All the HEN1 and HEN1 orthologs of green algae, lycophyte, bryophyte, gymnosperm, and angiosperm were clustered into a monophyletic group (Figure 8). Moreover, HEN1 orthologs of bryophyte also form a monophyletic group, which was sister to the SmHEN1-like protein of lycophyte (Figure 8). Except for the lineage-specific duplication events that occurred within species, including *Sphagnum fallax*, *Populus trichocarpa,* and Arabidopsis, other species only had single copy of HEN1 orthologs (Figure 8). This implied that the plant HEN1 might have a conserved role in the sRNA duplex methylation during plant evolution.

**Figure 9:**
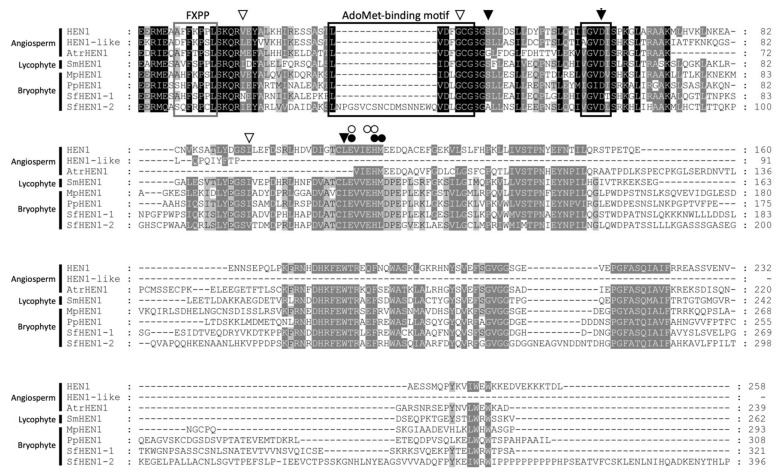
Multiple alignments of the MTase domain among different plant species. The arrowheads and asterisks indicate the SAM-binding residues and metal-binding residues, respectively. The black and grey boxes mark the AdoMet-binding motif and the FXPP motif, respectively.

We analyzed the conservation of the MTase domain of HEN1 among orthologs of lycophyte, bryophyte, and angiosperms. First, the sequence identity and similarity of the MTase domain were compared (Table 1 and Figure 9). The identity of the MTase domain was around 11.63~62.62% among plant species, and the identity showed 30.69~58.91% in bryophytes (Table 1). The wider difference of sequence identity resulted from the divergence of AtHEN1-like ortholog from other HEN1s. The AtHEN1-like ortholog contained a large deletion in the MTase domain (Figure 9). This implied that one copy of the Arabidopsis HEN1 paralog (AtHEN1) might maintain its major RNA MTase function, and another copy (AtHEN1-like) possibly had the same or divergent function after duplication. Then, we further investigated the conserved residues of the MTase domain. The alignment data indicated that some SAM-binding residues and metal-binding residues were highly conserved in the MTase domain (Figure 9). The FXPP motif in the N terminus of the MTase domain is essential for substrate recognition of eukaryotic HEN1 [40]. The alignment demonstrated that plant HEN1s displayed an FXP(P/S/C) sequence in the corresponding position (Figure 9). These data suggested that the HEN1 of plant species shared a common ancestor and had highly conserved sequence similarities, especially in the MTase domain.

## 4. Discussion

### 4.1. The Serology of HEN1

In this study, we were successful in generating α-AtHEN1 and α-MpHEN1 antibodies to detect recombinant proteins. In addition, the α-AtHEN1 can detect the exogenous HA-AtHEN1 in the *HA-AtHEN1* plant. However, α-AtHEN1 and α-MpHEN1 antibodies cannot detect the endogenous HEN1 in Arabidopsis and *M. polymorpha*. We suggested that this might have happened because of low level of endogenous protein expression or specific time and tissue-dependent expression of HEN1 in plant cells. In previous studies, it has been found that the expression pattern of HEN1 in *M. polymorpha* is developmental-stage-dependent, supporting our above hypothesis [41].

The 42% amino acid identity of the MTase domain between AtHEN1 and MpHEN1 could be the best possible explanation for α-AtHEN1 being able to show cross-reactivity toward the his-MpHEN1. However, the α-MpHEN1 antibody was generated by the full-length recombinant protein and hence became specific for his-MpHEN1 detection.

### 4.2. HC-Pro Has a Common HEN1 Inhibition Ability in Bryophyte and Angiosperm

The P1/HC-Pro is the first identified viral suppressor from the potyvirus species. The three most extensively studied HC-Pros, including TuMV, zucchini yellow mosaic virus (ZYMV), and tobacco etch virus (TEV), have demonstrated their suppression ability in plant [23,24,38,42,43]; however, the three different HC-Pros still hold differences. For instance, Leibman et al. (2011) demonstrated that HC-Pro that was purified from ZYMV-infected squash has sRNA-binding ability through the EMSA assay [44]. Moreover, Ruiz et al. (2015) have studied the sRNA derived from a TuMV-infected plant, and the sRNA profile indicated that virus derived sRNAs (visRNAs) can be co-immunoprecipited with HC-Pro, hypothesizing the sequestration of visRNAs by HC-Pro under TuMV infection [45]. However, our in vitro EMSA data indicated that recombinant GST-HC-Pro^R^ did not have sRNA duplex-binding ability. Hence, we hypothesize that purified HC-Pro from virus-infected plants might contaminate the other viral or host proteins that might help the sRNA to bind with HC-Pro.

Moreover, Hu et al. (2020) demonstrated that P1/HC-Pro of TuMV can trigger AGO1 degradation, but the P1/HC-Pro of ZYMV and TEV do not interfere with the AGO1 stability [42]. Another fine observation is that TuGR but not TuGK inhibits the methylation ability of AtHEN1, indicating the significance of arginine of FRNK motif in TuMV HC-Pro [23], unlike in case of ZYMV, where only the multiple amino acid substitutions completely abolished its suppression ability in transgenic Arabidopsis [43]. Concluding these observations, we propound that HC-Pros of different potyvirus interact with small RNA and different proteins of the silencing pathway in an exclusive manner, which individually needs to be studied.

The in vitro and in vivo HC-Pro^R^-mediated HEN1 inhibition assay demonstrated that HC-Pro^R^ alone is able to manifest the inhibitory effect on HEN1-mediated miRNA methylation without the assistance of P1. However, several studies have demonstrated HC-Pro alone is not sufficient enough to manifest the morphological and molecular defects; P1 and HC-Pro must work together to achieve the best gene silencing suppression as P1 enhances the suppression activity of HC-Pro [23,24,42,46,47,48,49]. Recent studies suggest that the P1s of TEV and PVY enhance the translation of respective P1/HC-Pro [30,42,50]. Considering this fact, we hypothesize that although P1 of TuMV does not directly alter the HEN1 methylation property, it might also enhance the amplification of HC-Pro, which eventually would promote the HEN1 inhibition.

To our surprise, the GST-HC-Pro^R^ inhibited the his-MpHEN1 activity in a dose-dependent manner in a very similar fashion to the way it did for his-AtHEN1, although *M. polymorpha*, a basal land plant, is by no means a host of TuMV, indicating that HC-Pro^R^ might interact with MpHEN1, which would require further experimental validations. Supporting this hypothesis, our alignment result showed that the HEN1 protein of both species is carrying largely conserved peptide sequences in different domains, especially the MTase domain. Further, it will be interesting to study the interactive pattern between viral HC-Pro and the plant’s HEN1 by targeting such sequences.

Previous studies demonstrated that unmethylated miRNAs will be poly-uridylated by HESO1/URT1, resulting in heterogenic sizes of miRNAs, which resembles the case of *hen1-1* [8] mutants. In contrast, oilseed rape mosaic tobamovirus (ORMV)-infected plants showed the heterogenic size of unmethylated miRNAs or siRNAs, suggesting that ORMV infection might suppress HEN1 activity, resulting in poly-uridylation [51]. Neither TuMV-infected Col-0 nor *P1/HC-Pro^R^* plants showed such a heterogenic size of miRNAs, suggesting the HC-Pro-mediated HEN1 inhibition might be different from ORMV. Together, these data provide shreds of evidence that silencing suppressors interfere with the modification stages of sRNA and also that apparently every silencing suppressor does not behave in the same fashion.

### 4.3. HEN1 Biology in Planta

The AtHEN1 and MpHEN1 contain two dsRBD domains that can specifically bind to an miRNA/miRNA* or siRNAs duplex but not single-stranded miRNAs or siRNAs. In contrast to plants, the HEN1 orthologs in animals can act on piRNAs or single-stranded siRNAs [40]. Moreover, not all small RNAs in animals are methylated by HEN1 orthologs. In *Caenorhabditis elegans*, the HEN1 of nematode (HENN1) specifically methylates Piwi-bound small RNAs in germlines [52]. The HEN1 ortholog of *Drosophila melanogaster* (DmHen1) acts on single-stranded piRNAs [15]. Mouse Hen1 (mHen1) is specifically expressed in testis and methylates piRNAs [14]. Combining these results, we can conclude that even these all belong to the kingdom Animalia but their methyltransferases have diverse substrate discrimination.

Interestingly, the HEN1 ortholog of *Chlamydomonas reinhardtii* also contains two dsRBD domains that can methylate miRNA/miRNA* and siRNA duplexes [3]. These pieces of evidence bring an interesting outcome that the sRNA duplex methylation in plant kingdom and algae is a conserved feature. The mechanism by which HEN1 orthologs distinguish between substrates has yet to be fully understood. In this study, the in vitro data showed AtHEN1 and MpHEN1, both belonging to the plantae, have strict substrate specificity and methylates-only RNA duplex, but not ssRNA [4,5,53]. In summary, these data suggest the structural and functional similarities between MpHEN1 and AtHEN1. Furthermore, it would be interesting to check the cross-substrate methylating efficiencies of different orthologous HEN1.

Tu et al. (2015) proposed that the 3′-end methylation protects miRNAs from uridylation and degradation in plants [8]. Moreover, the *hen1-1* mutant and *P1/HC-Pro^R^* plants showed a severe defective developmental phenotype that highlights the importance of HEN1-mediated miRNA methylation in plants. In *hen1-1* mutant, the accumulations of the most-tested miRNAs were unable to be detected or significantly reduced in abundance [54]. This means that some miRNAs are not totally eliminated in the *hen1* mutants. Additionally, some siRNAs remain detectable in the *hen1-4* mutant [10]. It suggested that the methylated or stabilized sRNA duplexes might be achieved by an alternative protein with similar abilities of HEN1. The HEN1-like heterologous protein is probably the candidate since it is the duplicated copy of HEN1 and has high sequence similarity with HEN1 (Table 1). Even though the Arabidopsis HEN1-like protein has a deletion in the C-terminus, several conserved amino acid residues for SAM binding and substrate binding still can be identified on the truncated MTase domain. Thus, we hypothesize that the presence of a HEN1-like protein in Arabidopsis might participate in a partial RNA interference mechanism through 3′-end 2′-*O*-methylation. However, the miRNAs in animals are not methylated, and such unmethylated miRNAs can still play a role in RNA silencing. These observations support the difference in miRNA turnover among plants and animals.

**Figure 10:**
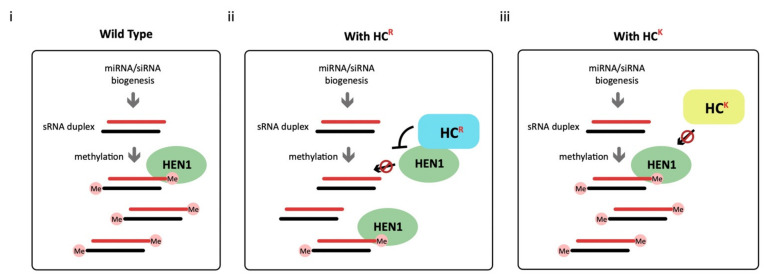
The working hypothesis for TuMV HC-Pro-mediated HEN1 inhibition for RNA silencing suppression in (**i**) wild-type healthy plants, (**ii**) TuGR-infected plants, and (**iii**) TuGK-infected plants.

We propose a working hypothesis for HC-Pro^R^-mediated HEN1 inhibition. In healthy plants, the HEN1 plays a role in miRNA/miRNA* and siRNA duplexes methylation for RNA silencing (Figure 10, panel i). In the TuGR-infected plants, the HC-Pro^R^ interacts and inhibits the HEN1, resulting in lesser sRNA duplexes methylation (Figure 10, panel ii). In contrast, the HC-Pro^K^ of TuGK loses the HEN1 interaction and inhibition potential; thus, the HEN1 still can bind the sRNA duplexes and effectuate the methylation activity of the plant’s RNA-silencing regulation (Figure 10, panel iii).

## 5. Conclusions

Here, we have found AtHEN1 to be more efficient, and its antibody showed cross-sensitivity toward MpHEN1, indicating the critical significance of the protein in higher plants. We also conclude that TuMV HC-Pro^R^, even without P1, interacts with and inhibits HEN1 methylation activity, and upon finding similar results with MpHEN1, we confirm that interaction is solely based on HEN1-HC-Pro and does not require any other subsidiary protein. In this study, both AtHEN1 and MpHEN1 remained excellent models to probe the substrate specificity of the protein as well as share conserved amino acid sequences reflecting their structural and functional similarities.

## Figures and Tables

**Table 1:** The similarity and identity of the MTase domains of plant HEN1 orthologs.

	Similarity	AtHEN1	AtHEN1-like	AtrHEN1	MpHEN1	PpHEN1	SfHEN1-1	SfHEN1-2	SmHEN1
Identity									
AtHEN1		-	51.98	65.09	61.88	56.43	54.70	37.37	69.80
AtHEN1-like		50.49	-	49.00	42.32	35.14	31.43	14.10	47.52
AtrHEN1		59.90	46.78	-	57.92	47.52	50.00	28.71	60.39
MpHEN1		54.95	39.35	52.22	-	59.40	63.11	42.32	63.86
PpHEN1		50.00	33.16	42.57	52.97	-	64.35	46.78	63.61
SfHEN1-1		48.51	30.44	44.80	57.17	58.91	-	47.27	58.66
SfHEN1-2		29.95	11.63	23.26	35.64	37.37	39.85	-	38.61
SmHEN1		62.62	44.80	54.20	57.17	56.43	52.47	30.69	-

## Data Availability

The data presented in this study are available on request from the corresponding author.
